# Risk stratification by regadenoson stress MRI in patients with known or suspected coronary artery disease

**DOI:** 10.1186/1532-429X-16-S1-P186

**Published:** 2014-01-16

**Authors:** Siddique A Abbasi, Ravi V Shah, Tomas Neilan, Bobak Heydari, Hoshang Farhad, Ron Blankstein, Michael Steigner, Michael Jerosch-Herold, Raymond Y Kwong

**Affiliations:** 1 Brigham and Women's Hospital, Boston, Massachusetts, USA; 2 Massachusetts General Hospital, Boston, Massachusetts, USA

## Background

With the expanding use of vasodilator stress CMR, there has been a parallel rise in the use of regadenoson as a pharmacological stress agent. To date, however, prognostic data for stress CMR has focused on the use of adenosine or dipyridamole while prognostic data regarding the use of regadenoson is limited. We therefore sought to identify the prognostic value of regadenoson vasodilator stress CMR on clinical events in patients with known or suspected coronary artery disease.

## Methods

We prospectively studied 346 consecutive patients clinically referred for CMR assessment of myocardial viability/ischemia over the course of 36 months. The CMR protocol consisted of: (1) cine SSFP functional imaging in multiple imaging planes, (2) stress perfusion imaging after administration of Regadenoson and a bolus injection of 0.1 mmol/kg intravenous gadolinium-DPTA; (3) late gadolinium enhancement imaging. Clinical follow-up was obtained via mailed questionnaire, review of electronic medical records, contact with the patients' cardiologist, and/or telephone contact with the patients. We used a primary composite endpoint of cardiovascular death or acute myocardial infarction.

## Results

A total of 346 patients were prospectively followed for a median time of 1.9 years. There were 7 cardiac deaths and 8 acute myocardial infarctions during the follow-up period. By univariable Cox regression modeling, the presence of inducible ischemia was associated with a near-7-fold increased risk of the primary outcome (HR = 6.95, P = 0.02). Patients with absence of inducible ischemia experienced a low annualized cardiac event rate of 0.6% per patient year. To the contrary, patients with inducible ischemia had an annualized rate of cardiac event of 3.2% per patient year.

## Conclusions

Regadenoson stress CMR is a prognostically useful examination in which a negative study is linked to a low risk of adverse cardiovascular outcomes in a prospective cohort of 346 patients followed for nearly 1½ years. In addition, a positive regadenoson stress CMR is associated with a significantly higher annualized rate of adverse cardiovascular events in patients referred for known or suspected CAD, even after adjustment for clinical and demographic characteristics.

## Funding

Astellas Pharma US, Inc.

**Table 1:** Univariable associations for cardiovascular death or acute myocardial infarction

Univariable Associations	
**Characteristic**	**Hazard Ratio (95% CI) LR χ2 P-value**

Age	1.01 (0.96-1.07) 0.25 0.62

Female	0.61 (0.12-3.13) 0.36 0.56

BMI	0.92 (0.80-1.06) 1.32 0.25

History of HTN	1.94 (0.35-10.6) 0.59 0.44

History of DM	2.16 (0.42-11.2) 0.85 0.35

History of Smoking	6.88 (1.54-30.8) 6.37 0.01

History of Dyslipidemia	0.70 (0.13-3.81) 0.17 0.68

LVEF	0.96 (0.93-1.00) 3.59 0.06

LVEDVI	1.01 (0.99-1.02) 0.31 0.58

LVESVI	1.01 (0.99-1.02) 0.98 0.32

Presence of LGE	8.59 (1.03-70.9) 3.94 0.04

Inducible Ischemia	6.95 (1.35-35.8) 5.36 0.02

Abbreviations: BMI = body-mass index, HTN = hypertension, DM = diabetes mellitus, LVEF = left ventricular ejection fraction, LVEDVI = left ventricular end diastolic volume index, LVESVI = left ventricular end systolic volume index, LGE = late gadolinium enhancement, LR = likelihood ratio.

